# Genetic insights into the mechanisms of proliferative glomerulonephritis

**DOI:** 10.1172/JCI183090

**Published:** 2024-08-01

**Authors:** Gentzon Hall

**Affiliations:** Department of Internal Medicine, Division of Nephrology, Duke University, Durham, North Carolina, USA.

## Abstract

Glomerular visceral epithelial cells (i.e., podocytes) are an essential component of the tripartite glomerular filtration barrier. Healthy podocytes are terminally differentiated cells with limited replicative capacity; however, inappropriate cell cycle reentry can be induced in podocytes by various injurious stimuli. In this issue of the *JCI*, Yamaguchi et al. report on a somatic mosaic gain-of-function mutation in the phosphatidylinositol-4,5-bisphosphate 3-kinase catalytic α subunit (p110α, encoded by *PIK3CA*). The study reveals that activating mutations of p110α can drive podocyte proliferation in *PIK3CA*-related overgrowth syndrome (PROS). They also showed that selective, small-molecule inhibitors of p110 may be useful for the treatment of proliferative glomerulonephritis.

## The podocyte

**Figure 1:**
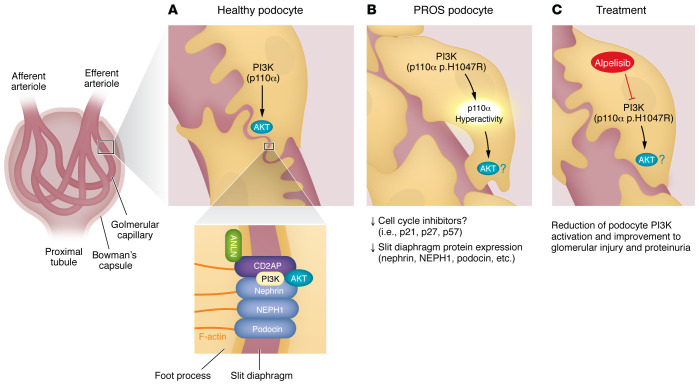
PI3K signaling in podocyte health and disease. (**A**) PI3K signaling is vital for the maintenance of podocyte identity, structure, survival, and function. The PI3K/AKT signaling pathway and its downstream effectors regulate actin cytoskeletal dynamics and survival signaling via slit diaphragm proteins such as CD2AP, Nephrin, NEPH1, and podocin. (**B**) Derangements of PI3K signaling can lead to podocyte dysfunction and the loss of slit diaphragm integrity. The somatic gain-of-function mutation, p.H1047R, drives hyperactivation of p110α, podocyte hyperplasia, dedifferentiation, loss of slit diaphragm protein expression, and the loss of glomerular integrity. (**C**) Yamaguchi et al. (21) demonstrated that BYL719 (alpelisib) effectively reduced podocyte PI3K activation and glomerular injury in a patient with proliferative glomerulonephritis caused by the p.H1047R mutation and in experimental models of proliferative glomerulonephritis.

Podocytes are specialized, postmitotic cells with essential functions in the maintenance of glomerular tuft architecture and ultrafiltration (Figure 1A) (1). As terminally differentiated cells, podocytes express high levels of the cell cycle inhibitory proteins p21, p27, and p57, which prevent initiation of the G_1_/S phase mitotic transition (Figure 1A) (2). This quiescent state is required in order to establish and maintain the podocyte’s unique cytoskeletal architecture (3). Processes that induce cell cycle reentry cause irreversible structural derangements, podocyte dedifferentiation, and can lead to cell death (Figure 1B) (4).

Mature podocytes are morphologically defined by an arborizing network of interdigitating actin-based pedicles (i.e., foot processes) (3). Foot processes attach to the glomerular tuft and provide the primary structural support for the filtration slit diaphragm (Figure 1A) (3). The slit diaphragm is a modified adherens junction comprised of a zipper-like assembly of cadherin and immunoglobulin-like proteins that span the interdigital spaces between foot processes (Figure 1A) (5). This approximately 40-nm-wide extracellular membrane serves as a heteroporous molecular sieve with size and charge selectivity properties for ultrafiltration (Figure 1A) (6). The slit diaphragm also serves as a dynamic signaling hub, transmitting extracellular information via transmembrane proteins like Nephrin, Podocin, and Neph1 to shape intracellular responses through key signal transduction pathways such as phosphoinositol-3 kinase (PI3K)/Ak strain transforming kinase (AKT) (Figure 1A) (7, 8).

## PI3K in signaling in podocytes

The cytoprotective actions of the PI3K/AKT signaling pathway have been extensively documented in the kidney (9). As in other epithelial cells, PI3K and its downstream effectors have been shown to regulate many vital physiologic processes in podocytes, including cell growth, survival, cytoskeletal regulation, motility, energy storage, and metabolism (Figure 1A) (10). AKT (also known as protein kinase B, PKB) is the principal downstream effector of PI3K (11). AKT is a ubiquitously expressed serine/threonine kinase with three functionally distinct isoforms (AKT1/PKB1, AKT2/PKB2, and AKT3/PKB3) (11). In 2013, Canaud et al. identified AKT2 as the primary mediator of podocyte cytoprotective signaling in response to stress (Figure 1A) (12). Other studies have shown that AKT physically associates with slit diaphragm proteins such as Nephrin and CD2-associated protein (CD2AP) to establish nodes of antiapoptotic signaling and cytoskeletal regulation (Figure 1A) (13–15). Despite these cytoprotective functions, it is well known that perturbations of PI3K/AKT signaling in chronic kidney disease are associated with deleterious processes such as epithelial-mesenchymal transformation, endoplasmic reticulum stress, pathologic hypertrophy, fibrosis, hyperplasia, and apoptosis (Figure 1B) (16). In proliferative podocytopathies such as idiopathic collapsing focal segmental glomerulosclerosis (cFSGS) and HIV-associated nephropathy (HIVAN), we and others have shown that inappropriate upregulation of cell cycle regulatory proteins, such as human telomerase (hTERT) and anillin (ANLN), drive podocyte proliferation, in part, through hyperactivation of PI3K/AKT signaling (17, 18). Since AKT is a potent negative regulator of the cyclin-dependent kinase inhibitors p21, p27, and p57, it is possible that hyperactivation of AKT in cFSGS and HIVAN promotes podocyte proliferation via suppression of these key cell cycle inhibitors (Figure 1B) (19, 20). Although the precise mechanisms are still to be determined, these observations suggest a potential role for dysregulated PI3K/AKT signaling in the pathobiology of podocyte proliferation (Figure 1B). In this issue of the *JCI*, Yamaguchi et al. describe the effects of a somatic mosaic gain-of-function mutation (p.H1047R) in the phosphatidylinositol-4,5-bisphosphate 3-kinase catalytic α subunit (p110α, encoded by *PIK3CA*) on the development of proliferative glomerulonephritis in a patient with *PIK3CA*-related overgrowth syndrome (PROS), providing evidence for the role of PI3K hyperactivation in proliferative podocytopathy and highlighting selective p110α inhibition as a potential rational therapy for the disease (Figure 1, B and C) (21).

## PROS and kidney disease

PROS is a heterogeneous collection of diseases resulting from postzygotic activating mutations in p110α (22). The clinical presentation of the disease varies with the timing of the somatic mutation during embryonic development and the degree of tissue mosaicism (22). The disease can manifest as isolated or multiple lesions, including sporadic or mosaic overgrowth of adipose, skeletal, muscle, brain, vascular, or lymphatic tissue (22). Skin involvement can also occur, manifesting as epidermal nevi and hyper- or hypopigmented lesions (22). PROS mutations are among the most common mutations identified in cancer and there are currently no targeted therapies for the disease (22).

Activating p110α mutations predominantly occur in hotspots in the helical and kinase domains, but can occur in other regions of the protein (23). The disease-causing p.H1047R mutation (c.3140A>G substitution in PIK3CA) in p110α occurs in the C-terminal kinase domain and leads to hyperactivation of the PI3K/AKT signaling pathway and abnormal tissue growth (23). In 2018, Canaud et al. first described a 29-year-old male patient (Patient 1) with PROS caused by the p.H1047R mutation (24). Although renal complications are rare in PROS, the patient’s condition included a complex proliferative glomerulonephritis, prompting a focused exploration of the pathogenic effects of the p.H1047R mutation in podocytes (21, 24). Yamaguchi et al. now report the findings of their follow-up study on Patient 1 (21). A diagnostic kidney biopsy showed cFSGS with extensive fibrosis, pseudocrescent formation, tubular dilation with casts, and inflammatory cell infiltration. No immune deposits were observed. Immunofluorescence studies showed activation of the PI3K/AKT pathway in podocytes and in situ PCR, using primers designed to amplify the c.3140A>G substitution, detected the mutation in podocytes (21). The presence of the p.H1047R p110α mutation was confirmed by droplet digital PCR in microdissected glomeruli at a mosaic fraction of 8% (21). Empiric treatment with BYL719 (alpelisib), a selective small-molecule inhibitor of p110α with approximately 250- to 1000-fold reduced inhibitory activity for p110β, p110γ, and p110δ isoforms, improved the patient’s kidney function and proteinuria (21, 25). Subsequent evaluation of BYL719 in four experimental models of proliferative glomerulonephritis (i.e., p110α hyperactivation [R26StopFLP110*]), HIV-associated nephropathy (Tg26), and lupus glomerulonephritis (*MRL-lpr* and NZBWF1/J) demonstrated similar improvements in glomerular injury, kidney function, and proteinuria reduction (21). Notably, BYL719 attenuated immune cell activation and cytokine production in both experimental models of lupus nephritis (i.e., *MRL-lpr* and NZBWF1/J) (21). Yamaguchi et al. attributed this to the suppression of p110δ activity in lymphocytes (21). These findings highlight the potentially pleiotropic benefits of BYL719 in the treatment of proliferative glomerulonephritis and reveal a role for aberrant PI3K activation as a driver of podocyte hyperplasia (21).

## Therapeutically targeting podocyte proliferation

There are currently no targeted therapies for proliferative podocytopathy, likely owing to our poor understanding of the mechanisms of disease. While it is clear that several injurious stimuli can provoke podocyte proliferation (e.g., genetic, viral infection, malignancy, ischemic disease, autoimmune disease, toxic exposure, etc.), these insights have not revealed discrete molecular targets for treatment of the disease. Standard-of-care therapies focus on controlling hypertension, proteinuria reduction, and blockade of the renin angiotensin system. In some instances, potent immunosuppressive agents are also used (e.g., glucocorticoids, calcineurin inhibitors, antimetabolites, B cell–depleting monoclonal antibodies), which often fail to stabilize disease progression and may provoke off-target effects. Importantly, Yamaguchi et al. provide evidence of a proliferative podocytopathy caused by a somatic gain-of-function mutation in p110α (21). Insights from this study may inform our understanding of the pathogenic mechanisms of podocyte cell cycle reentry and provide a basis for broader exploration of PI3K pathway antagonists in the treatment of disease. What remains unknown are the precise mechanisms of p110α-mediated cell cycle reinitiation, the contributions of AKT isoforms to podocyte proliferation in human disease, and the potential therapeutic utility of targeting other PI3K pathway intermediates for the treatment of proliferative glomerulonephritis.

## Version 1 08/01/2024

Electronic publication
